# Relationships between work-related factors and musculoskeletal health with current and future work ability among male workers

**DOI:** 10.1007/s00420-017-1216-0

**Published:** 2017-03-25

**Authors:** J. S. Boschman, A. Noor, R. Lundström, T. Nilsson, J. K. Sluiter, M. Hagberg

**Affiliations:** 1 Coronel Institute of Occupational Health, Academic Medical Center, Amsterdam Public Health Research Institute, University of Amsterdam, Amsterdam, The Netherlands; 2 Occupational and Environmental Medicine, Sahlgrenska Academy and University Hospital, University of Gothenburg, Gothenburg, Sweden; 3 Department of Radiation Sciences, Umeå University, Umeå, Sweden; 4 Occupational and Environmental Medicine, Umeå University, Umeå, Sweden

**Keywords:** Cohort studies, Occupational health, Musculoskeletal disease, Work, Hand strength, Occupations

## Abstract

**Purpose:**

The purpose was to increase job-specific knowledge about individual and work-related factors and their relationship with current and future work ability (WA). We studied cross-sectional relationships between mental demands, physical exertion during work, grip strength, musculoskeletal pain in the upper extremities and WA and the relationships between these variables and WA 11 years later.

**Methods:**

We used a dataset of a prospective cohort study (1997–2008) among employees of an engineering plant (*n* = 157). The cohort was surveyed by means of tests and written questions on work demands, musculoskeletal health, WA score (WAS; 0–10), and mental and physical WA. Spearman correlation coefficients and logistic regression analysis were used.

**Results:**

Among manual workers, we found weak correlations between grip strength and current and future physical WA. We did not find predictors for future poor WA among the manual workers. Among the office workers, we found that musculoskeletal pain was moderately and negatively related to current WAS and physical WA. More handgrip strength related to better future WAS and physical WA. Musculoskeletal pain (OR 1.67 *p* < 0.01) and lower handgrip strength (OR 0.91 *p* < 0.05) predicted future poor WA among office workers.

**Conclusions:**

Our results showed cross-sectional and longitudinal relationships between musculoskeletal health and work ability depending on occupation. However, the present implies that predicting work ability in the far future based on health surveillance data is rather difficult. Testing the musculoskeletal system (grip strength) and asking workers’ about their musculoskeletal health seems relevant when monitoring work ability.

## Introduction

The concept of sustainable employability and work ability has gained growing attention over the years (van der Klink et al. 2016). Preventing health-related job loss, that is ensuring job retention, is considered an increasingly important outcome in occupational health research (Viikari-Juntura and Burdorf 2011). This is because consistently low birth rates and higher life expectancy are causing a transition towards a much older population structure (Eurostat 2015; WHO 2011). As a result, the proportion of people of working age is shrinking, while the relative number of those retired is growing (Eurostat 2015). The need for workers who will continue to work at an older thus increases as a consequence of having to maintain national production at the same level.

The concept of work ability reflects a balance between work demands and the individual resources of a worker to meet those demands (Ilmarinen et al. 1997). Work-related factors that are associated with poor work ability include high mental work demands, lack of autonomy and high physical work load (van den Berg et al. 2009). Individual factors related to poor work ability include musculoskeletal pain (Lindegard et al. 2014), poor musculoskeletal capacity (van den Berg et al. 2009) and older age (van den Berg et al. 2009). Two cross-sectional studies reported lower handgrip strength to be associated with lower work ability especially in workers exposed to hand–arm vibration (Edlund et al. 2012; Rentzsch et al. 2015).

Factors associated with work ability have been studied by numerous scholars (Gould and Ilmarinen 2008; van den Berg et al. 2009). Most research done in this field is based on cross-sectional data and some studies report prospective results (Boschman et al. 2015, 2014; von Bonsdorff et al. 2011a). If occupational health professionals wish to encourage sustainable work ability they should be provided with knowledge about the factors that are related to future work ability. From a preventive point of view, intervening at an early stage is the most beneficial strategy. However, little is known about the time frame: can we predict work ability 2, 5, 10 or even 20 years from now? Moreover, what parameters would be the best predictors, and do they depend on the occupation?

In addition to this knowledge gap, more knowledge on suitable tests measuring predictors of future work ability would be valuable. For example, musculoskeletal capacity as a pure personal factor has been operationalized as trunk flexion and extension endurance or strength; balance; upper extremities endurance; lower extremities endurance; trunk flexibility; and hand grip strength (Nygård et al. 1991; Pohjonen 2001). Composite scores have also been reported; for example, a score based on 11 tests that were carried out by a physiotherapist among healthy individuals and those with coronary artery disease or low-back pain (Eskelinen et al. 1991). Especially for workers in occupations with high physical demands, it seems relevant to take into account musculoskeletal capacity in the search for useful predictors of work ability.

The SUNDS cohort, a unique cohort of Swedish workers at an engineering plant that has been followed since 1987 (Nilsson et al. 1989), allows the assessment of the issues outlined above. The last follow-up measurement dates from 2008 and included work ability as a parameter. The unique data of the SUNDS cohort allow us to assess relationships between work factors, aspects of health, grip strength—as an indicator of musculoskeletal capacity—and work ability over a longer period of time. Literature on factors that predict work ability based on longitudinal data that span 10 years or more is rather limited. Studies have found that working conditions, such as the psychosocial work environment (Feldt et al. 2009) and physical work demands (von Bonsdorff et al. 2011b), including hand-transmitted vibration (Bovenzi et al. 2015), and individual factors such as musculoskeletal pain, depressive symptoms (Punakallio et al. 2014) and mental work strain (von Bonsdorff et al. 2011b), are related to an adverse development in work ability. Based on these previous findings, we designed this study. We chose 11 years as follow-up based on practical considerations and as we believed that this time frame is relevant for practice.

This cohort study focused on predictors that could be measured within the scope of preventive occupational healthcare. The aim of this study was to analyse the relationship between workers’ health surveillance outcomes and work ability after 11 years between 2 types of occupations: office workers and manual workers. Our main hypothesis was that higher mental work demands, higher physical exertion during work, and musculoskeletal health problems would be indicative of future poor work ability, in comparison to colleagues with lower work demands and better musculoskeletal health. Furthermore, we hypothesized that better handgrip strength would be related to better future work ability, particularly among manual workers.

We analysed longitudinal data collected from workers at a Swedish engineering plant, with a follow-up of 11 years. For both the total group of workers, as well as the manual workers and office workers separately, we asked:

1. What is the cross-sectional relationship between work demands (mental demands, physical exertion), musculoskeletal health (grip strength, musculoskeletal pain in the upper extremity) and work ability?
2. What is the relationship between work demands and musculoskeletal health and future work ability?

## Methods

### Study population

For this study we used data from a prospective cohort study (1997–2008) among employees of an engineering plant in Sweden. The Regional Ethical Review Board in Umeå (Dnr 07-161M) approved the original study. The cohort consisted of male office workers and male manual workers. At the time of inclusion they were all full-time employees at the engineering plant, which manufactured products associated with pulp and paper. The subjects were recruited from the plant’s payroll list in January 1987 and January 1992. An upper age limit of 55 years was set as a criterion for inclusion to guarantee a sufficiently long period of follow-up in which the workers would still be employed. Among the manual workers (*n* = 182) were welders, grinders, turners, and steel platers. The office workers (*n* = 89) included salesmen, managers, engineers, secretaries, and administrative clerks.

Follow-up measurements were conducted in 1997 and 2008. The occupation in 2008 was used to categorize the study participant as ‘manual worker’ or ‘office worker’. By checking the exposure to working with vibrating hand tools (yes/no), it was verified whether the worker was correctly considered a ‘manual worker’ or ‘office worker’. At the 2008 follow-up 228 subjects (18% loss from baseline) remained in the cohort. Among them, 119 were working at the same workplace (the engineering plant), 43 had either changed workplace or become self-employed, 53 had taken the pension and 2 were job seekers (for 11 no information was available). The participants that were lost to follow-up did not differ from those not lost to follow-up regarding age and exposure [reported by Sandén et al. (2010)]. For this study, we included only those individuals who were working and answered the work ability score question (112 manual workers and 45 office workers).

### Measurements

#### Work ability

Work ability was measured using questions from the WAI (Ilmarinen 2007). The concept of work ability can be defined as the ability of a worker to perform his/her job, taking into account the specific work demands, individual health condition and mental resources. The WAI is a widely disseminated, valid, reliable and commonly used tool for measuring work ability and the study of El Fassi et al. indicated that using the single-item approach does not deteriorate the validity of the work ability information collected. The level of convergent validity was satisfactory (rs = 0.63) (El Fassi et al. 2013). The first question of the WAI is also referred to as Work Ability Score (WAS), which measures current general work ability compared to lifetime best work ability (0 = not able to work, 10 = best ever) (Ahlstrom et al. 2010). Current physical and mental work ability compared to lifetime best was measured using the relevant questions from the WAI. Answer categories ranged from 0 = very poor to 5 = very good.

#### Job demands

Mental job demands were measured in 1997 and 2008 with four questions that addressed work speed and quantity (work fast; work hard; whether the work is excessive; whether there is enough time to do the job) and were based on the demands described by Karasek (Karasek 1979). The items were scored on a 4-point scale (1 = never, 2 = seldom, 3 = sometimes, 4 = often). We recoded items such that high scores always had a negative interpretation. A total score was calculated by adding the scores from the respective items. A higher score reflected high mental demands.

Physical exertion was measured in 1997 and 2008 with a Borg scale for self-reported exertion related to the work over the last 7 work weeks on a scale from 0 to 14 (0 = very easy, 14 = very strenuous) (Borg 1982).

#### Musculoskeletal health

Musculoskeletal health was operationalized by musculoskeletal complaints and grip strength. In 1997 and 2008, musculoskeletal complaints for each bodily region were measured with the question ‘Do you currently experience pain, aching, numbness or discomfort in the following body region?’ (yes/no).

In 1997 and 2008, the maximal grip strength of the hand was measured by a hand dynamometer. The maximum static handgrip force was assessed at baseline by a laboratory-developed handle, equipped with a Bofors strain gauge bridge and an electronic amplifying unit. In 2008, assessments were performed with a Jamar^®^ hand dynamometer (Sammons Preston Royal, Model 5030J1, Inspected by, and with a certificate of calibration: 5349, 1084; Bolingbrook Illinois). The assessment procedures were comparable.

The participant was positioned with their elbow at a 90° angle and the wrist in a neutral position, with the underarm supported by the table. They were then instructed to grasp the handle 3 times with each hand at 10-s intervals, starting with the dominant hand (Mathiowetz et al. 1985). The maximum grip strength (*N*) was used in the present study.

The questionnaire further comprised information on age and occupation.

### Analysis

Data were eligible for analysis when the question on WAS was answered (dependent variable). Descriptive statistics were presented as a percentage, mean, or median, with standard deviation (SD) and/or range. SAS 9.4 for Windows software was used to analyse the data. Statistical significance was set at an alpha level of 0.05. A visual check for the normality of WAS (primary outcome) and the independent variables was performed by inspection of the histogram graphs. Based on this inspection we decided to use parametric or non-parametric tests to analyse the data. The data were analysed for the whole group and separately for the manual workers and office workers. Due to missing values, the number of respondents can differ.

Musculoskeletal complaints of the neck, shoulder, arms and hands were dichotomized into ‘Having any upper extremity complaints’ and ‘Not having any upper extremity complaints’. Musculoskeletal complaints, handgrip strength, mental demands and physical exertion in 1997 and 2008 were then related to WAS, and to physical and mental work ability in 2008 using Spearman’s rho. The Spearman correlations were interpreted as follows: 0.00–0.19 ‘very weak’; 0.20–0.39 ‘weak’; 0.40–0.59 ‘moderate’; 0.60–0.79 ‘strong’; 0.80–1.0 ‘very strong’ (Weir 2011).

We then dichotomized WAS into ‘Moderate or poor work ability’ (i.e. ‘poor’ work ability) (WAS ≤8) and ‘Good work ability’ (WAS 9 or 10). Physical and mental work ability were dichotomized into ‘poor work ability’ (very poor, poor, moderate) and ‘good work ability’ (good, very good) (Gould and Ilmarinen 2008). The correlations of independent variables that showed a statistically significant relationship with the work ability outcomes were included in the logistic regression analysis. Three univariate regression analyses were performed for the total group of workers: one that included the data from 1997, one that included the 2008 data and one that included the difference between 2008 and 1997. For continuous independent variables, we calculated odds ratios (OR), for binary variables prevalence ratios (PR) and for variables representing the difference we calculated relative risks (RR).

## Results

There was no statistically significant difference in WAS between office workers (8.4 ± 1.26) and manual workers (8.0 ± 1.79) (*p* = 0.17) in 2008. In total, 68 out of 157 workers (44%) reported good or excellent work ability (i.e. a ‘9’ or ‘10’). None of the office workers reported a WAS of five or lower, and among the manual workers this only applied to eight workers (4%). In total, 89% of the workers reported good physical work ability, 95% reported good mental work ability.

In 1997, the participants were 39 years of age on average: the office workers (*n* = 45) were 43- years- old on average (25–75th percentiles: 38–48), the manual workers 38 years (25–75th percentiles: 30–46) (*n* = 112). The characteristics of the study participants are presented in Table 1.

**Table 1:** Description of the characteristics of office workers (*n* = 58) and manual workers (*n* = 140) at an engineering plant participating in a follow-up study in both 1997 and 2008

Variable	1997			2008		
	Total	Office workers	Manual workers	Total	Office workers	Manual workers
*N*	157	45	112	157	45	112
Age (years) median	40	45	39	51	56	50
Range	16–54	24–53	16–54	27–65	35–64	27–65
Work ability score						
Median				8	8	8
Range				0–10	6–10	0–10
Good physical work ability (%)				89	96	86
Good mental work ability (%)				95	98	94
Physical exertion						
Median	6	4	7	5	3	7
Range	0–12	0–9	0–12	0–11	0–6	0–11
Mental demands						
Median	10	10	9	9	9	9
Range	2–15	2–15	4–14	4–15	4–15	4–13
Musculoskeletal pain upper extremities (%)	32	36	30	38	24	43
Handgrip left (*N*)						
Mean	47	47	48	53	50	54
Range	27–72	27–65	32–72	28–78	29–62	28–78
Handgrip right (*N*)						
Mean	50	49	50	55	54	56
Range	29–75	29–68	29–75	28–80	40–71	28–80

### Cross-sectional correlations 2008

The outcome variables and independent variables are described in Table 1. When we analysed the data of the whole population, we found no statistically significant correlations between mental demands and WAS, and mental demands and physical or mental work ability (Table 2). We found ‘very weak’ correlations between musculoskeletal pain (*r* = −0.17, *p* = 0.05) and both left and right handgrip strength and WAS (*r* = 0.17, *p* < 0.05) and physical work ability (*r* = 0.17–0.18, *p* < 0.05) when measured at the same time (2008, Table 2). Physical exertion correlated weakly (*r* = −0.19, *p* < 0.05) with physical work ability, indicating a weak trend, such that the higher the perceived physical exertion, the lower the self-reported physical work ability.

**Table 2:** Results of the correlation analysis based on the cross-sectional data for manual and office workers in 2008

Variable	Work ability score		Physical work ability		Mental work ability	
	Correlation coefficient	*p* value	Correlation coefficient	*p* value	Correlation coefficient	*p* value
Physical exertion						
Total population	−0.08	>0.05	**−0.25**	<**0.05**	**−0.16**	<**0.05**
Manual workers	**−**0.11	>0.05	**−**0.18	>0.05	**−**0.09	>0.05
Office workers	**−**0.03	>0.05	**−**0.30	>0.05	**−**0.35	>0.05
Mental demands						
Total population	**−**0.01	>0.05	**−**0.09	>0.05	**−**0.16	>0.05
Manual workers	0.07	>0.05	**−**0.03	>0.05	**−**0.09	>0.05
Office workers	**−**0.26	>0.05	**−**0.35	>0.05	**−**0.36	>0.05
Musculoskeletal pain upper extremities						
Total population	**−**0.17	0.05	**−**0.14	>0.05	**−**0.09	>0.05
Manual workers	**−**0.07	>0.05	**−**0.03	>0.05	**−**0.05	>0.05
Office workers	**−0.50**	<**0.01**	**−0.45**	<**0.05**	**−**0.15	>0.05
Handgrip left						
Total population	**0.17**	<**0.05**	**0.17**	<**0.05**	0.14	>0.05
Manual workers	0.17	>0.05	0.18	>0.05	0.13	>0.05
Office workers	0.24	>0.05	0.29	>0.05	0.31	>0.05
Hand-grip right						
Total population	**0.17**	<**0.05**	**0.18**	<**0.05**	0.13	>0.05
Manual workers	0.17	>0.05	**0.22**	<**0.05**	0.14	>0.05
Office workers	0.23	>0.05	0.12	>0.05	0.13	>0.05

Bold printed figures are statistically significant findings

The analyses of the data for both occupational groups separately showed a statistically significant but ‘weak’ correlation between right handgrip strength and physical work ability among the manual workers (*r* = 0.22, *p* < 0.05). Among the office workers, we found statistically significant moderate correlations between musculoskeletal pain and both WAS (*r* = **−**0.50, *p* < 0.01) and physical work ability (*r* = **−**0.45, *p* = 0.01).

In summary, when measured at the same time point, more hand-grip strength was correlated with better WAS and better physical work ability among manual workers, whereas musculoskeletal complaints of the upper extremities were negatively related to WAS and physical work ability among office workers. Physical exertion was only found to be statistically significantly correlated with physical work ability when the data of the total group were analysed. No variables were statistically significantly correlated with mental work ability.

### Correlations with work ability 11 years later (’97–’08)

For the group as a whole, we found ‘weak’ correlations between both left and right hand-grip strength measured in 1997 and WAS (*r* = 0.21, *p* < 0.05) and physical work ability (*r* = 0.25–0.30, *p* < 0.05) measured in 2008 (Table 3). The analyses of the manual workers and office workers separately, showed statistically significant weak correlations between handgrip strength in 1997 (*r* = 0.24–0.33, *p* < 0.05) and physical work ability in 2008 among the manual workers, but not the office workers. Among the office workers, left handgrip strength in 1997 was found to be correlated with mental work ability in 2008 (*r* = 0.32, *p* < 0.05).

**Table 3:** Results of the correlation analysis based on the individual and work-related factors in 1997 and work ability in 2008 (total population)

Variable	Work ability score		Physical work ability		Mental work ability	
	Correlation coefficient	*p* value	Correlation coefficient	*p* value	Correlation coefficient	*p* value
Physical exertion						
Total population	**−**0.03	>0.05	**−**0.13	>0.05	**−**0.16	>0.05
Manual workers	0.07	>0.05	0.05	>0.05	**−**0.07	>0.05
Office workers	**−**0.09	>0.05	**−**0.30	0.05	**−**0.21	>0.05
Mental demands						
Total population	**−**0.07	>0.05	**−**0.05	>0.05	**−**0.13	>0.05
Manual workers	**−**0.07	>0.05	**−**0.07	>0.05	**−**0.18	>0.05
Office workers	**−**0.08	>0.05	**−**0.05	>0.05	**−**0.09	>0.05
Musculoskeletal pain upper extremities						
Total population	**−**0.02	>0.05	0.00	>0.05	0.12	>0.05
Manual workers	0.08	>0.05	0.06	>0.05	0.16	>0.05
Office workers	**−**0.23	>0.05	**−**0.12	>0.05	0.05	>0.05
Handgrip left						
Total population	**0.21**	<**0.05**	**0.25**	<**0.05**	0.17	>0.05
Manual workers	0.18	>0.05	0.24	>0.05	0.10	>0.05
Office workers	0.22	>0.05	0.29	>0.05	**0.32**	<**0.05**
Handgrip right						
Total population	**0.21**	<**0.05**	**0.30**	<**0.01**	0.17	0.05
Manual workers	0.21	0.05	**0.33**	<**0.01**	0.19	>0.05
Office workers	0.21	>0.05	0.24	>0.05	0.18	>0.05

Bold printed figures are statistically significant findings

In summary, greater handgrip strength measured in 1997 was weakly related to better work ability in 2008 among both the manual workers (physical work ability) and the office workers (mental work ability).

### Predictors of future poor work ability

The univariate logistic regression analyses for the whole population showed no statistically significant associations between musculoskeletal pain in the upper extremities, mental demands or physical exertion and poor WAS. However, higher left handgrip strength in 1997 and 2008 had a protective association with poor WAS in 2008 [OR 0.96 (95% CI 0.92–1.00, *p* < 0.05)].

Among office workers, we found that musculoskeletal pain in the upper extremities in both 1997 [PR 1.67 (95% CI 1.02–2.74, *p* = 0.05)] and 2008 [PR 2.06 (95% CI 1.35—3.14, *p* < 0.01)] was associated with poor WAS in 2008 (Tables 4, 5). Furthermore, among office workers, we also found a statistically significant protective effect of left handgrip strength in 1997 and the risk of poor WAS in 2008 [RR 0.91 (95% CI 0.83–1.00, *p* = 0.04)]. We did not find statistically significant associations for the manual workers.

**Table 4:** Results of the logistic regression analyses based on the cross-sectional data for manual and office workers in 2008

Variable		Poor work ability score (0–8)		
		Risk estimate	95% CI	*p* value
Physical exertion		PR		
Manual workers		1.15	0.79–1.66	>0.05
Office workers		n.a		
Mental demands		PR		
Manual workers		0.79	0.54–1.15	>0.05
Office workers		0.79	0.39–1.61	>0.05
Musculoskeletal pain upper extremities	PR			
Manual workers		0.97	0.70–1.35	>0.05
Office workers		**2.06**	**1.35–3.14**	<**0.01**
Handgrip left		OR		
Manual workers		0.98	0.93–1.02	>0.05
Office workers		0.94	0.87–1.02	>0.05
Handgrip right		OR		
Manual workers		0.98	0.95–1.02	>0.05
Office workers		0.95	0.87–1.03	>0.05

*n.a*. not applicable as there were no office workers with high physical exertion

Bold printed figures are statistically significant findings

**Table 5:** Results of the logistic regression analyses based on the individual and work-related factors in 1997 and WAS in 2008 for manual and office workers

Variable	Poor work ability score (0–8)		
	Risk estimate	95% CI	*p* value
Physical exertion	PR		
Manual workers	0.88	0.64–1.21	>0.05
Office workers	1.13	0.49–2.62	>0.05
Mental demands	PR		
Manual workers	0.96	0.66–1.41	>0.05
Office workers	0.72	0.72–1.96	>0.05
Musculoskeletal pain upper extremities	PR		
Manual workers	1.04	0.74–1.47	>0.05
Office workers	**1.67**	**1.02–2.74**	<**0.01**
Handgrip left	OR		
Manual workers	0.97	0.93–1.02	>0.05
Office workers	**0.91**	**0.83–1.00**	<**0.05**
Handgrip right	OR		
Manual workers	0.97	0.92–1.02	>0.05
Office workers	0.96	0.89–1.04	>0.05

Bold printed figures are statistically significant findings

In summary, among the office workers we found a protective effect of higher grip strength and an increased risk of future poor WAS in relation to musculoskeletal pain. We did not find mental demands or physical exertion to be associated with poor WAS.

## Discussion

In the present study we found that musculoskeletal health, operationalized as handgrip strength and musculoskeletal pain in the upper extremities, correlated with work ability when the measures were taken at the same point in time and when these measures were separated by 11 years. Furthermore, we found that musculoskeletal health is a predictor of future poor work ability. Our job-specific findings showed that: (1) musculoskeletal pain in the upper extremities was negatively and moderately correlated with work ability of office workers when measured at the same point in time, while hand-grip strength of manual workers was positively and weakly correlated with work ability; (2) hand-grip strength was for both office and manual workers, related with work ability 11 years later. Neither changes in physical exertion nor musculoskeletal health were predictive of future poor work ability.

### Comparison with previous studies

Our results are in line with previous cross-sectional findings by Edlund et al. (2012) and Nygård et al. (1991). They also found weak correlations (*r* = 0.31–0.32) between handgrip strength and work ability. Edlund et al. (2012) studied 47 individuals with vascular and/or neurological symptoms in the hands, while the results of Nygård et al. (1991) were based on a study among 65 women in different occupations. We assessed if and how the type of occupation affected this relationship. Therefore, we analysed data of occupations that are—in general—regarded as more physically demanding (manual workers) or more mentally demanding (office workers). We expected handgrip strength to be more strongly related to physical work ability among the manual workers, as having good handgrip strength might be regarded as a job-specific feature. Among this group of manual workers and office workers, this assumption was not confirmed. Similar results were found by Pohjonen (2001), who studied 132 female home care workers with a 5-year follow-up and did not find hand-grip strength to be a statistically significant predictor of work ability, despite this occupation potentially being regarded as more physically demanding.

Furthermore, Nygård et al. (1991) did not find a cross-sectional correlation for the men in their study. As indicated by the results above, the scientific evidence regarding the relationship between handgrip strength and work ability is not unanimous. Although there is evidence that higher scores on handgrip strength is associated with lower rates of mortality and lower risk of morbidity, the predictive value is not that distinct for work ability.

Pohjonen (2001) underlined the usefulness of physical fitness tests for occupational health professionals in predicting work ability. The findings of our present study accord with this idea to some extent, but we recommend the use of both tests and questionnaires to operationalize musculoskeletal health as predictors of current work ability and future work ability. Based on our findings measuring handgrip strength and asking about musculoskeletal complaints of the upper extremities seem to be useful.

### Interpretation of the results

The SUNDS cohort provided us with a unique opportunity to study mental and physical work demands and musculoskeletal health as predictors of work ability in the distant future. We hypothesized that both the individual and work-related factors would affect perceived work ability among the employees of an engineering plant, at the current time and in the future. These hypotheses were only partly confirmed.

One explanation for not finding associations with future work ability might be a lack of contrast in WAS in the study population. Previous research by Ilmarinen et al. (1997) showed a significant decrease in the work ability index over 11 years and found that 25% of the study population had poor work ability, while excellent work ability was rare. These findings do not match ours: our study population seemed to have a considerably better work ability and only very few workers could be said to have poor work ability. This could be an indication of bias due to the healthy worker effect. Furthermore, the separate logistic regression analyses for each occupational group showed that musculoskeletal pain in the upper extremities (measured in 1997) was predictive of poor work ability/WAS in 2008 among the office workers, but not among the manual workers. This finding might reflect a healthy worker effect among the manual workers since musculoskeletal pain is a known risk factor for poor work ability (Miranda et al. 2010), disability and early retirement (Alavinia and Burdorf 2008). Information on reasons for employment termination, job changes related to underlying health status or types of inactive work status (Buckley et al. 2015) was not gathered in the present cohort. Therefore, it remains impossible to better characterize and control for underlying health status (Buckley et al. 2015).

Another explanation, which complements the previous above, might be a lack of variation in work demands and musculoskeletal health. Our study population consisted of a cohort of workers employed at one workplace, possibly reducing the variation in predictors. Nevertheless, we included very distinct occupational groups: both the manual workers and the office workers. Therefore, we do not think a lack of variation in predictors can be seen as an important factor for not finding associations between mental demands, physical exertion and musculoskeletal pain, and work ability 11 years later.

### Strengths and limitations

Gathering data in a longitudinal study with a long-term follow-up can be challenging for several reasons. In the present study, different hand dynamometers were used for the measurements in 2008 and 1997. We found positive mean differences in handgrip strength from 1997 to 2008 and attributed this to the use of different hand dynamometers. As handgrip strength declines with increasing age (Frederiksen et al. 2006), a mean increase from baseline to follow-up was not expected or plausible. This systematic error did not affect the analyses or results in the present study as differences were only assessed at the individual, not at the group, level.

Furthermore, over time, the working environment, working methods and tools at the engineering plant changed. In the present study this was reflected in the decrease in physical exertion experienced by more than half of the workers: although they had aged, they perceived their work as less strenuous. Technological improvements and the fact that they performed skilled manual work could be the cause of this. Over all, this makes it difficult to assess the influence of changes in work demands over time.

The results of the present study reflect measurements from a limited group of workers and, therefore, need to be interpreted with caution and confirmed in larger studies that can take confounding factors into account. Although this study is exploratory and conclusions regarding the causality of the relationship between predictors and work ability cannot be assumed, it adds to our knowledge of predictors of work ability in the far future. This knowledge is crucial in preventive occupational health care aimed at sustainable employability.

### Implications

Workers’ health surveillance is one such preventive strategy (International Labour Organization 1998). One of the main goals of workers’ health surveillance is to promote the sustainable work ability of employees (Sluiter et al. 2013). The WAI and WAS (Schouten et al. 2015) are frequently used to assess work ability for surveillance purposes (Boschman et al. 2011). Most studies on factors related to work ability report results based on cross-sectional measurements and it remains difficult to assess their implications for practice. The present longitudinal study, however, implies that predicting work ability in the far future is rather difficult. Handgrip strength might seem a promising measure to include in workers’ health surveillance programs, but more questions need to be answered first. As the correlations are weak, it would be interesting to see if and how an improvement in grip strength relates to an improvement in work ability. Furthermore, it remains difficult to use grip strength in a surveillance program, as no cut-off values are available that indicate a grip strength too low for sufficient work ability. Research addressing these issues might be a useful next step. Thus far, our results add to the knowledge that it remains difficult to predict workers’ future health (Roelen et al. 2016) and work ability based on health surveillance data.
